# AM966, an Antagonist of Lysophosphatidic Acid Receptor 1, Increases Lung Microvascular Endothelial Permeability through Activation of Rho Signaling Pathway and Phosphorylation of VE-Cadherin

**DOI:** 10.1155/2017/6893560

**Published:** 2017-02-27

**Authors:** Junting Cai, Jianxin Wei, Shuang Li, Tomeka Suber, Jing Zhao

**Affiliations:** ^1^Acute Lung Injury Center of Excellence, Division of Pulmonary, Asthma, and Critical Care Medicine, Department of Medicine, University of Pittsburgh, Pittsburgh, PA 15213, USA; ^2^Third Affiliated Hospital of Xiangya Medical School, Changsha, Hunan 410013, China; ^3^Department of General Surgery, The First Affiliated Hospital of Dalian Medical University, Dalian, Liaoning 116011, China

## Abstract

Maintenance of pulmonary endothelial barrier integrity is important for reducing severity of lung injury. Lysophosphatidic acid (LPA) regulates cell motility, cytoskeletal rearrangement, and cell growth. Knockdown of LPA receptor 1 (LPA1) has been shown to mitigate lung injury and pulmonary fibrosis. AM966, an LPA1 antagonist exhibiting an antifibrotic property, has been considered to be a future antifibrotic medicine. Here, we report an unexpected effect of AM966, which increases lung endothelial barrier permeability. An electric cell-substrate sensing (ECIS) system was used to measure permeability in human lung microvascular endothelial cells (HLMVECs). AM966 decreased the transendothelial electrical resistance (TEER) value immediately in a dose-dependent manner. VE-cadherin and f-actin double immunostaining reveals that AM966 increases stress fibers and gap formation between endothelial cells. AM966 induced phosphorylation of myosin light chain (MLC) through activation of RhoA/Rho kinase pathway. Unlike LPA treatment, AM966 had no effect on phosphorylation of extracellular signal-regulated kinases (Erk). Further, in LPA1 silencing cells, we observed that AM966-increased lung endothelial permeability as well as phosphorylation of VE-cadherin and focal adhesion kinase (FAK) were attenuated. This study reveals that AM966 induces lung endothelial barrier dysfunction, which is regulated by LPA1-mediated activation of RhoA/MLC and phosphorylation of VE-cadherin.

## 1. Introduction

Lysophosphatidic acid (LPA) is a bioactive phospholipid that contributes to the pathogenesis of numerous fibrotic diseases, including pulmonary, hepatic, skin, and renal fibrosis [1–3]. Upon binding to its high-affinity G protein-coupled receptors (LPA1–6) and coupling to different downstream G proteins (G*α*i/o, G*α*q, and G*α*12/13) [4, 5], LPA exerts multiple biological effects, including cell proliferation, migration, cytoskeletal rearrangement, and cell survival [6–8]. Studies have shown that LPA levels in bronchoalveolar lavage (BAL) fluid increase in idiopathic pulmonary fibrosis (IPF) patients [2, 9, 10]. The LPA-LPA1 pathway plays a crucial role in the development of pulmonary fibrosis via mediating fibroblast growth and recruitment [2]. AM966 is a highly selective LPA1 antagonist [11], which inhibited LPA-stimulated intracellular calcium release and LPA-induced chemotaxis in vitro and reduced lung injury and fibrosis induced by bleomycin in vivo [12]. Based on these findings, AM966 has gained considerable academic and industry attention as a treatment for IPF [12–15]. A relative compound BMS-986202 (previously AM152) has completed phase 1 clinical trials in 2011, and the phase 2 clinical trials of another structurally related molecular BMS-986020 is completed in 2016 [14, 16, 17].

Maintenance of pulmonary endothelial barrier integrity is of great importance in healthy lungs. Impaired microvascular endothelial barrier function leads to the infiltration of blood proteins and circulating cells into the tissues underlying vessels, which is related to lung injury [18, 19]. The endothelial cell-cell junctional complex that controls paracellular permeability is composed of adherens junctions, tight junctions, and desmosomes. Adherens junctions are a major part of the complex, in which VE-cadherin, an endothelium-specific component of adhesion proteins, controls both adherens junctions and endothelial barrier integrity [20, 21]. Inflammatory stimuli, such as thrombin and endotoxin, induce phosphorylation of VE-cadherin and redistribution of VE-cadherin from cell-cell junctions to the cytoplasm, thus increasing vascular permeability [20]. In addition to adherens junctions, myosin light chain- (MLC-) mediated cytoskeletal remodeling also majorly contributes to gap formation and endothelial barrier dysfunction [22, 23]. A small GTPase, RhoA, and its downstream signaling molecule, Rho kinase, regulate MLC phosphorylation and induce stress fiber, thereby causing cell retraction and endothelial leak [24, 25].

LPA has been shown to increase lung and corneal epithelial barrier integrity [26], while studies about the role of LPA in endothelial barrier function are controversial. Some earlier studies reported a protective role of LPA in endothelial cell barrier integrity [27, 28], while more recent studies demonstrated increased vascular leakage after LPA exposure [2, 29–31]. Furthermore, genetic deletion of LPA1 has been shown to induce embryonic lethality [32], indicating LPA1 signaling pathway is of great importance in healthy beings. Given the characteristic of AM966 in selectively inhibiting LPA1 receptor, we hypothesized that AM966 has a role in regulating endothelial barrier function. In the present study, we show that AM966 increases permeability in human lung microvascular endothelial cells (HLMVECs) by activation of Rho signaling pathway and phosphorylation of VE-cadherin. Our findings reveal an unexpected effect of AM966, which raises a caution for using AM966 as an antifibrotic medicine in the future.

## 2. Materials and Methods

### 2.1. Reagents and Cell Culture

Human lung microvascular endothelial cells (HLMVECs, Lonza) were cultured at 37°C in an atmosphere of 5% CO_2_ with EGM-2 medium (Lonza) containing 25 mL FBS (5%), 0.5 mL hEGF, 2.0 mL hFGF-*β*, 0.5 mL VEGF, 0.5 mL ascorbic acid, 0.2 mL hydrocortisone, 0.5 mL R3-IGF-1, and 0.5 mL gentamycin. Phospho (T18/S19)-MLC, MLC, antibodies, and cell lysis buffer were obtained from Cell Signaling. Phospho (Y658)-VE-cadherin antibody was purchased from Invitrogen. VE-cadherin antibody was from Santa Crus Biotechnology. *β*-Actin antibody, scrambled siRNA, LPA1 siRNA, and LPA were from Sigma Aldrich. LPA1 antibody was obtained from Proteintech. AM966 was from Apex Bio. Horseradish peroxidase-conjugated goat anti-mouse and anti-rabbit secondary antibodies, ECL kit, and SDS-PAGE for western blotting were purchased from Bio-Rad Laboratories, Inc. For immunostaining, anti-mouse Alexa-488, anti-rabbit Alexa-568, and DAPI were from Invitrogen. Transfection reagent FuGENE HD was from Promega. All other reagents were of analytical grade.

### 2.2. siRNA Transfection

SiRNAs and Lipofectamine RNAi MAX reagent (Invitrogen) were diluted separately in Opti-MEM medium and then incubated together for 5 min at room temperature before adding to the cell culture. Analysis of the transfected cells was performed 72 h later.

### 2.3. Protein Extraction and Western Blot Analysis

Cells were lysed in lysis buffer. Samples were loaded with equal amounts of total protein (20 *μ*g) and separated by 4–15% SDS-PAGE gels and then transferred to nitrocellulose membranes. Membranes were blocked with 5% nonfat milk and then incubated with specific primary antibodies, followed by secondary antibodies. The membranes were developed using chemiluminescence detection system.

### 2.4. Immunofluorescence Staining

HLMVECs were cultured in glass-bottomed dishes and were fixed with 3.7% formaldehyde in phosphate-buffered saline (PBS) for 20 min. After blocking with 1% bovine BSA in TBST for 30 min, cells were exposed to VE-cadherin for 1 h. Then, anti-rabbit Alexa-488 secondary antibody was applied for 1 h. F-Actin was immunostained with Alexa-568 Phalloidin. Nuclei were detected with DAPI. Immunofluorescent cell imaging was performed using a Zeiss LSM 510 confocal microscope.

### 2.5. Measurement of TEER by Electrical Cell-Substrate Impedance Sensing System (ECIS)

HLMVECs grown on gold electrodes and experiments were conducted only on wells with steady-state transendothelial electrical resistance. Resistance changes were monitored in real time using ECIS (Applied Biophysics) with 4000 Hz. TEER values from each microelectrode were pooled at discrete time points and plotted versus time as the mean ± SEM.

### 2.6. RhoA Activity Assay

HLMVECs were treated with AM966 (1.0 *μ*M, 15 and 30 minutes) or thrombin (1 U/mL, 30 minutes). Guanosine triphosphate- (GTP-) bound active Rho was predicated by following the manufacturer's instructions (Rho Activation Assay Kit, Millipore). The amount of activated RhoA is determined by a western blotting using a RhoA specific antibody.

### 2.7. Statistical Analysis

Statistical analysis was carried out by one-way ANOVA with post hoc test or Student's *t*-test, with a *p* value of < 0.05 considered indicative of significance.

## 3. Results

### 3.1. AM966 Increases Barrier Permeability and Gap Formation between Lung Microvascular Endothelial Cells

**Figure 1:**
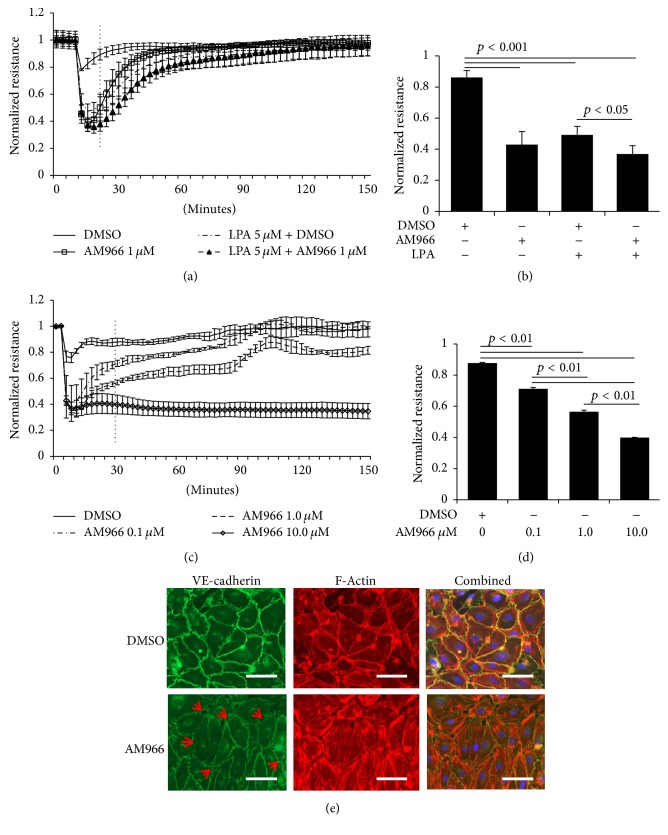
AM966 increases permeability in HLMVECs. (a) Confluent HLMVECs were plated on gold electrodes and TEER changes were monitored in real time using ECIS. After baseline resistance was stable, DMSO, AM966 (1.0 *μ*M), LPA (5 *μ*M), or AM966 + LPA was added to each well. The TEER tracing represents pooled data (±SEM) from 3 independent experiments. (b) The electrical resistance during indicated time period (a) was quantified and statistical analysis was performed. (c) Confluent HLMVECs were plated on gold electrodes and TEER changes were monitored in real time using ECIS. After baseline resistance was stable, different doses of AM966 (0.1, 1.0, or 10.0 *μ*M) were added to each well. The TEER tracing represents pooled data (±SEM) from 3 independent experiments. (d) The resistance in response to AM966 treatments during indicated time period (c) was quantified and statistical analysis was performed. (e) HLMVECs (~100% confluence) were grown on a glass bottom coverslip and serum deprived for 3 h, and then the cells were treated with DMSO or AM966 (1 *μ*M) for 30 min. Immunofluorescence staining of VE-cadherin (green), F-actin (red), and nuclei (blue) was examined by a Zeiss LSM 510 confocal microscope. Scale, 15 *μ*m. Paracellular gaps are marked by arrows. Shown are representative images from three independent experiments.

It has been reported that LPA increases lung endothelial barrier permeability, while the effect of LPA1 antagonist on lung endothelial barrier integrity has not been reported. Our initial studies examined the effect of AM966 on HLMVECs barrier function using ECIS system, a highly sensitive system to measure endothelial cell monolayer integrity and permeability. Figures 1(a) and 1(b) show that AM966 rapidly reduces TEER in 15 min after treatment. The resistance returned to baseline within 2 h. This effect was similar to LPA treatment. The combination of LPA1 agonist (LPA) and antagonist (AM966) had no further reduction of TEER. These data suggest that both AM966 and LPA increase HLMVECs permeability and delays barrier integrity recovery time. VE-cadherin is a major junction protein, which controls endothelial barrier integrity. Next, we examined whether the effect of AM966 is dose-dependent. As shown in Figures 1(c) and 1(d), AM966 reduces TEER in a concentration-dependent manner. The TEER recovered (0.1 and 1.0 *μ*M AM966) within 2 h, while it remained in a low level with 10 *μ*M AM966 stimulation. Further, we examined the effect of AM966 on VE-cadherin expression on cell surface. As shown in Figure 1(e), VE-cadherin is primarily localized on cell-cell junctions. However, AM966 (1 *μ*M, 30 min) causes paracellular gap formation and less VE-cadherin staining on cell-cell junctions. F-Actin staining shows that A966 increases stress fibers in HLMVECs (Figure 1(e)). Taken together with Figure 1, these data indicate that AM966 treatment leads to reduction of VE-cadherin expression on the cell-cell junction, increasing stress fibers, and gap formation, thus disrupting lung microvascular barrier integrity.

### 3.2. AM966 Activates RhoA and Increases Phosphorylation of MLC in HLMVECs

**Figure 2:**
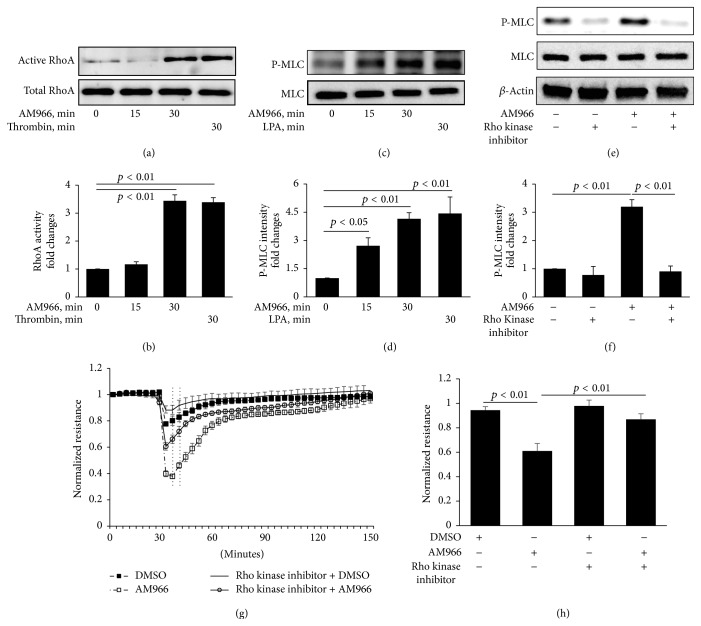
AM966 induces RhoA activity and phosphorylation of MLC. (a) Confluent HLMVECs were starved 3 h and treated with AM966 (1.0 *μ*M) and thrombin (1 U/mL) for indicated time periods. Activated RhoA was immunoprecipitated from total lysates by following the manufacturer's instructions (Rho Activation Assay Kit, Millipore). The amount of activated RhoA is determined by a western blot using a RhoA specific antibody. (b) Analysis of activated RhoA by densitometry of the results in (a) were performed by Image J software (*n* = 3), and statistical analysis was shown. (c) Confluent HLMVECs were treated with AM966 (1.0 *μ*M) or LPA (5 *μ*M) for indicated time periods after 3 h starvation. Cell lysates were immunoblotted with phospho-MLC (P-MLC) and total MLC antibodies. (d) Analysis of P-MLC by densitometry of the results in (c) was performed by Image J software (*n* = 3), and statistical analysis was shown. (e) Serum starved confluent HLMVECs were pretreated with Rho kinase inhibitor (10 *μ*M) for 1 h and then incubated with DMSO or AM966 (1.0 *μ*M) for an additional 30 min. Lysates were immunoblotted with P-MLC, total MLC, and *β*-actin antibodies. (f) Analysis of P-MLC by densitometry of the results in (e) was performed by Image J software (*n* = 3), and statistical analysis was shown. Shown are representative blots from three independent experiments. (g) Confluent HLMVECs were plated on gold microelectrodes and pretreated with 10.0 *μ*M Rho kinase inhibitor for 1 h and then stimulated by 1.0 *μ*M AM966 or DMSO. The TEER tracing represents pooled data (±SEM) from 3 independent experiments. (h) The resistance in response to AM966 treatments during indicated time period (g) was quantified and statistical analysis was performed.

A primary mechanism of cellular contraction is the actin-myosin cross-bridge interaction. Rho/Rho kinase plays a pivotal role in direct or indirect phosphorylation of MLC [23]. Based on AM966 induction of stress fibers, we hypothesized that AM966 activates RhoA in HLMVECs. To examine whether RhoA/Rho kinase pathway contributes to AM966-increased lung endothelial permeability, we first examined the RhoA activity after AM966 treatment and found that AM966 stimulation activated RhoA (Figures 2(a) and 2(b)). Thrombin, an agent well known to activate Rho pathway, was used as a positive control (Figures 2(a) and 2(b)). Further, the effect of AM966 on MLC phosphorylation was determined. As shown in Figures 2(c) and 2(d), AM966 induced phosphorylation of MLC in a time-dependent manner, while the effect was inhibited by Rho kinase inhibitor (Figures 2(e) and 2(f)). The effect of AM966 on phosphorylation of MLC was similar to the effect by LPA (Figures 2(c) and 2(d)). To examine whether RhoA activation is involved in AM966-reduced TEER, HLMVECs were treated with Rho kinase inhibitor prior to AM966 addition. As shown in Figures 2(g) and 2(h), AM966-reduced TEER was significantly attenuated by Rho kinase inhibitor. These data suggest that RhoA activation and MLC phosphorylation play a critical role in AM966-induced lung endothelial barrier disruption.

### 3.3. AM966 Increases Phosphorylation of VE-Cadherin

**Figure 3:**
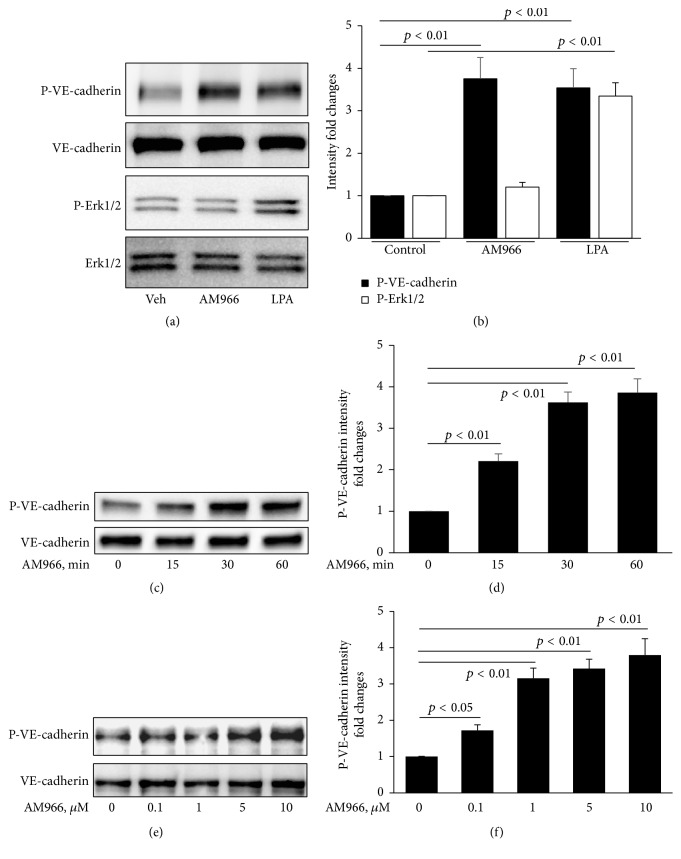
AM966 induces phosphorylation of VE-cadherin. (a) Confluent HLMVECs were treated with AM966 (1.0 *μ*M) or LPA (5.0 *μ*M) for 30 min after 3 h starvation. Cell lysates were immunoblotted with phospho-VE-cadherin (P-VE-cadherin), total VE-cadherin, phospho-Erk1/2 (P-Erk1/2), and total Erk1/2 antibodies. (b) Analysis of P-VE-cadherin and P-Erk1/2 by densitometry of the results in (a) was performed by Image J software (*n* = 3), and statistical analysis was shown. (c) Confluent HLMVECs were treated with AM966 (1.0 *μ*M) for indicated time after 3 h starvation. Cell lysates were immunoblotted with P-VE-cadherin and total VE-cadherin antibodies. (d) Analysis of P-VE-cadherin by densitometry of the results in (c) was performed by Image J software (*n* = 3), and statistical analysis was shown. (e) Confluent HLMVECs were treated with AM966 (0 to 10.0 *μ*M) for 30 min after 3 h starvation. Cell lysates were immunoblotted with P-VE-cadherin and total VE-cadherin antibodies. (f) Analysis of P-VE-cadherin by densitometry of the results in (e) was performed by Image J software (*n* = 3), and statistical analysis was shown. Shown are representative blots from three independent experiments.

It has been well known that tyrosine phosphorylation of VE-cadherin reduces endothelial cell-cell junctions [20]. We therefore explored the effect of AM966 on phosphorylation of VE-cadherin. Figures 3(a) and 3(b) depict a similar level of increased VE-cadherin phosphorylation after treatment with AM966 or LPA, while increase in phosphorylation of Erk1/2 was only observed in LPA treated cells. Our and others' previous studies have shown that G*α*i regulates LPA-induced Erk1/2 phosphorylation [8, 33, 34]. This data suggests that AM966 induced phosphorylation of VE-cadherin is not through G*α*i pathway. Furthermore, AM966 induced phosphorylation of VE-cadherin is in both a time- and concentration-dependent manner (Figures 3(c)–3(f)). These data support the hypothesis that AM966 reduces lung endothelial barrier integrity through modulation of VE-cadherin phosphorylation and reduction of VE-cadherin expression on the cell-cell junctions in HLMVECs.

### 3.4. AM966 Induces Phosphorylation of VE-Cadherin and Endothelial Barrier Disruption through LPA1

**Figure 4:**
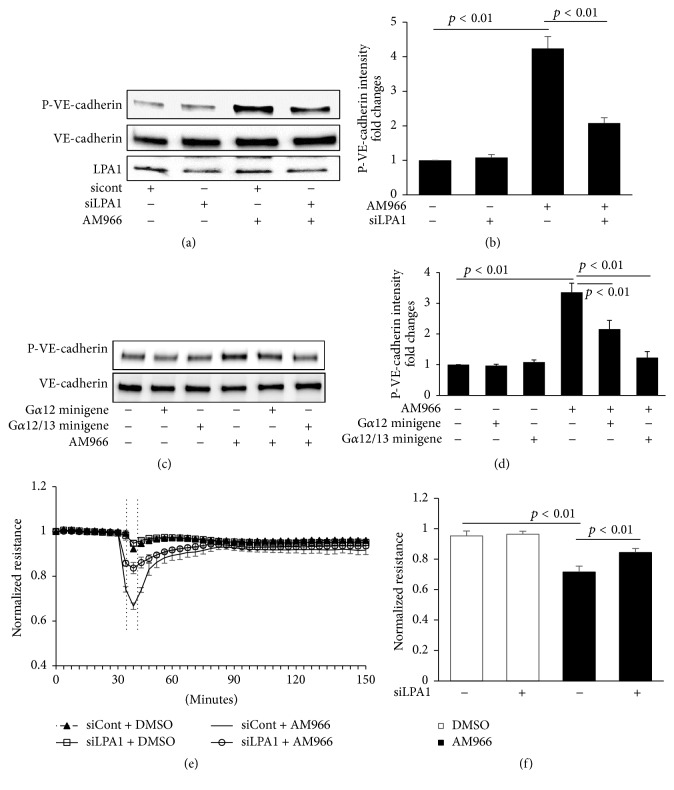
LPA1 is required for AM966-induced phosphorylation of VE-cadherin. (a) HLMVECs (~70% confluent) were transfected with LPA1 siRNA (siLPA1) or control (sicont) for 72 h, and then cells were treated with AM966 (1.0 *μ*M) for 30 min. Cell lysates were immunoblotted with P-VE-cadherin, total VE-cadherin, and LPA1 antibodies. (b) Analysis of P-VE-cadherin by densitometry of the results in (a) was performed by Image J software (*n* = 3), and statistical analysis was shown. (c) HLMVECs (~70% confluent) were transfected with minigenes encoding G*α*12 or G*α*13 peptide for 24 h, and then cells were treated with AM966 (1.0 *μ*M) for 30 min. Cell lysates were immunoblotted with P-VE-cadherin and total VE-cadherin antibodies. (d) Analysis of P-VE-cadherin by densitometry of the results in (c) was performed by Image J software (*n* = 3), and statistical analysis was shown. Shown are representative blots from three independent experiments. (e) HLMVECs (~70% confluent) transfected with LPA1 siRNA (siLPA1) or control (sicont) were plated on gold microelectrodes and then cells were treated with AM966 (1.0 *μ*M) or DMSO. The TEER tracing represents pooled data (±SEM) from 3 independent experiments. (f) The resistance in response to AM966 treatments during indicated time period (e) was quantified and statistical analysis was performed.

Though AM966 is a competitive antagonist of the LPA1 receptor, here we show that AM966 induces biological effects in HLMVECs including phosphorylation of VE-cadherin and MLC, activation of RhoA, and reduction of TEER. Thus, we hypothesized that AM966-mediated barrier disruption is through binding to LPA1 receptor. Downregulation of LPA1 expression with siRNA significantly attenuated the AM966-induced phosphorylation of VE-cadherin as shown in Figures 4(a) and 4(b). LPA1 is coupling to different downstream G proteins (G*α*i/o, G*α*q, and G*α*12/13) to regulate multiple biological effects. To investigate the involvement of G*α*12/13, we transiently transfected HLMVECs with the minigene vectors (Cue Biotech, Chicago, IL) encoding a unique peptide that specifically blocks the receptor/G protein interface. As shown in Figures 4(c) and 4(d), G*α*12/13 minigene transfection resulted in attenuation of AM966-induced phosphorylation of VE-cadherin. These results indicate that G*α*12/13 are essential for AM966 reduction of lung endothelial barrier integrity. The functional importance of LPA1 receptor in barrier regulation is also measured using ECIS system. LPA1 siRNA transfection markedly attenuated AM966-reduced TEER (Figures 4(e) and 4(f)). These data indicate that AM966-induced barrier disruption in HLMVECs is mediated by LPA1/G*α*12/13 pathways including phosphorylation of VE-cadherin.

### 3.5. AM966-Induced Phosphorylation of VE-Cadherin Is Not FAK-Dependent

**Figure 5:**
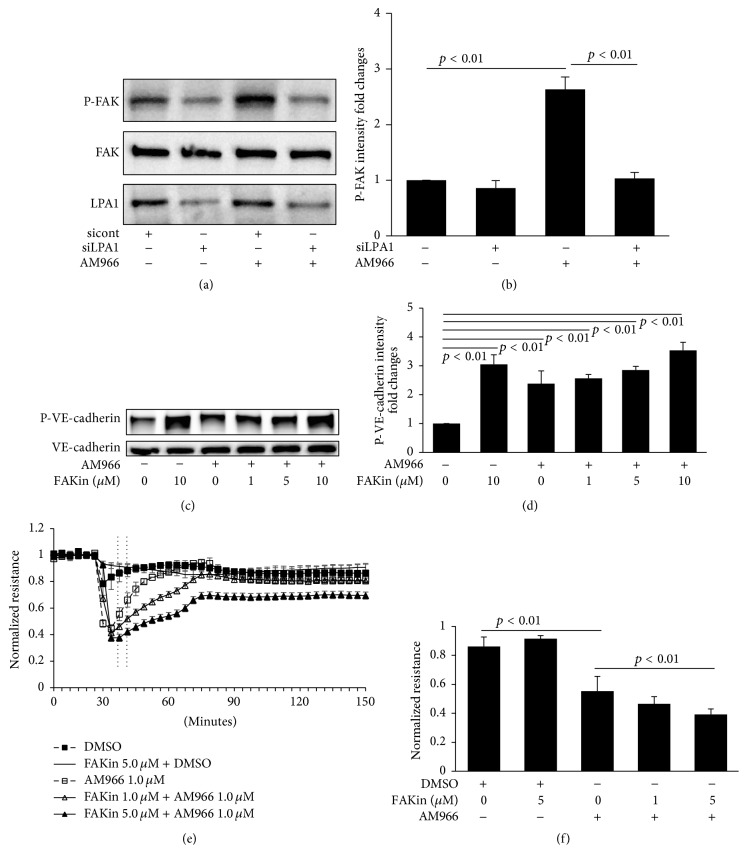
AM966-induced phosphorylation of VE-cadherin is not FAK-dependent. (a) HLMVECs (~70% confluent) transfected with siLPA1 or sicont were treated with AM966 (1.0 *μ*M) or DMSO for 30 min. Cell lysates were immunoblotted with phosphospecific FAK (P-FAK), total FAK, and LPA1 antibodies. (b) Analysis of P-FAK by densitometry of the results in (a) was performed by Image J software (*n* = 3), and statistical analysis was shown. (c) Confluent HLMVECs were pretreated with FAK kinase inhibitor (0 to 10.0 *μ*M) for 1 h, and then cells were treated with DMSO or AM966 (1.0 *μ*M) for an additional 30 min. Cell lysates were immunoblotted with P-VE-cadherin and total VE-cadherin antibodies. (d) Analysis of P-VE-cadherin by densitometry of the results in (c) was performed by Image J software (*n* = 3), and statistical analysis was shown. Shown are representative blots from three independent experiments. (e) Confluent HLMVECs were plated on gold microelectrodes and pretreated with FAK inhibitor (1.0 or 5.0 *μ*M) for 1 h and then stimulated by 1.0 *μ*M AM966 or DMSO. The TEER tracing represents pooled data (±SEM) from 3 independent experiments. (f) The resistance in response to AM966 treatments during indicated time period (e) was quantified and statistical analysis was performed.

It has been shown that VE-cadherin phosphorylation is mediated by focal adhesion kinase (FAK) [35]. We observed that AM966 induced phosphorylation of FAK; the effect was attenuated by downregulation of LPA1 (Figures 5(a) and 5(b)), suggesting that FAK is a downstream signal molecule of AM966/LPA1. However, the FAK kinase inhibitor alone increased VE-cadherin phosphorylation, and it enhanced AM966-induced FAK phosphorylation in a dose-dependent manner (Figures 5(c) and 5(d)). The functional of FAK inhibitor in barrier regulation is also measured using ECIS system. FAK inhibitor promoted AM966-reduced TEER in a dose-dependent manner (Figures 5(e) and 5(f)). This data suggests that FAK is not the kinase that induces tyrosine phosphorylation of VE-cadherin in response to AM966 stimulation in HLMVECs.

## 4. Discussion

IPF is a chronic and progressive lung disorder, which may result from abnormal lung repair and remodeling [19]. LPA/LPA1-mediated fibroblast proliferation and migration are implicated in the pathogenesis of IPF; thus, targeting LPA1 pathway is a new potential therapeutic strategy to treat IPF. AM966 is a highly selective oral LPA1 antagonist exhibiting an antifibrotic property [12]. However, the biological effects of AM966 have not been investigated. Maintenance of pulmonary endothelial barrier integrity is of great importance for reducing severity of lung injury. The current study demonstrates that AM966 induces lung microvascular endothelial barrier disruption in vitro through modulation of VE-cadherin phosphorylation and cytoskeletal rearrangement. These effects are mediated by LPA1. This study is the first to reveal that AM966 may exhibit endothelial barrier disruption properties.

Controversial results regarding the effects of LPA on endothelial barrier integrity have been reported during the past two decades. Decreased endothelial permeability by platelet-derived LPA was first observed by Alexander et al. [28]. Further, albumin-bound LPA was found to form an active complex that increases electoral resistance across endothelial cells [36]. The effect of LPA on reduction monolayer permeability was also observed in Schlemm's canal cells [37] and in the late angiogenesis [27]. However, more recent studies demonstrated increased vascular leakage after LPA exposure [18, 38, 39], and LPA1 regulates the phenotype [39–41]. Deregulation of adherens and tight junctions [29, 42, 43], calcium release [44], and Rho-mediated cytoskeletal rearrangement [25, 30, 45, 46] contribute to LPA-induced endothelial barrier disruption. In our study, we provide evidence to show not only LPA but also LPA1 antagonist, AM966, increases permeability immediately and reversibly.

A previous study has shown that when giving 10 mg/kg of AM966 orally to nonfasted mice, plasma AM966 reached a peak concentration of 9 *μ*M within 1 h [12], which indicates that the AM966 in our experiments were in the biological range. However, the study shows that oral administration of AM966 protects lung vascular leak after 7 days of bleomycin challenge [12]. This controversial conclusion may be due to species specificity. Pan et al. have shown that molecular regulation of gene expression of p-selection, an adhesion receptor, is different from mouse and human endothelial cells [47].

**Figure 6:**
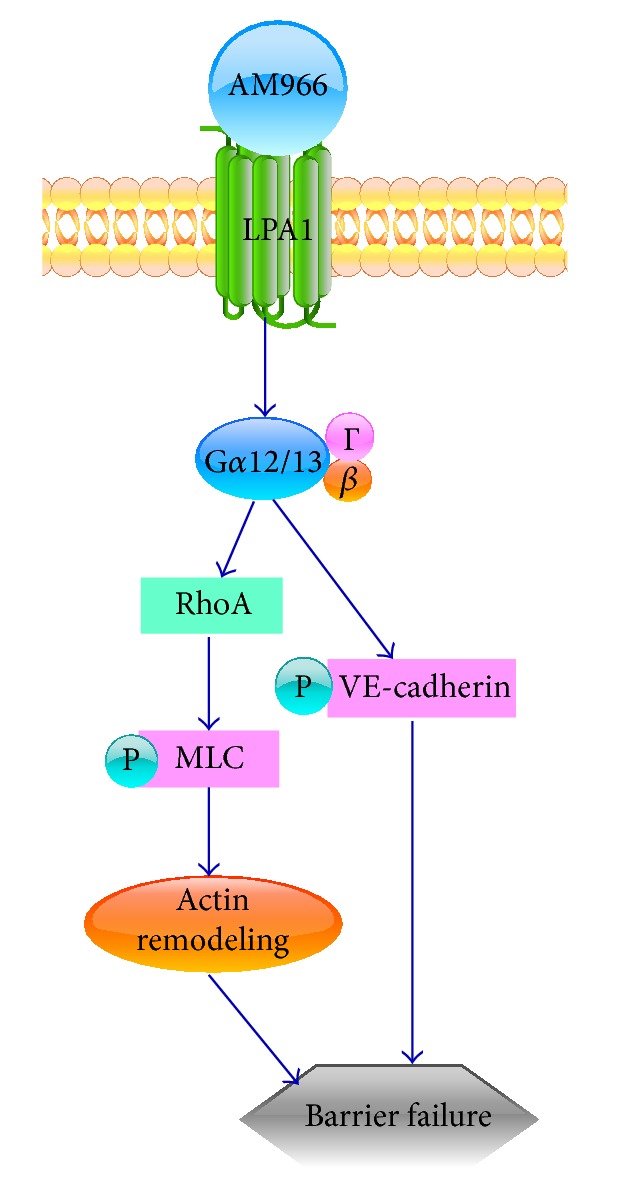
AM966 reduces pulmonary microvascular cell barrier integrity through LPA1/G*α*12/13-mediated phosphorylation of MLC and VE-cadherin. AM966 is ligated to LPA1 and triggers RhoA/Rho kinase pathway, thereby increasing MLC phosphorylation and cytoskeletal rearrangement. AM966 induces phosphorylation of VE-cadherin. Therefore, AM966 increases lung endothelial permeability.

Accumulating findings have shown that, with thrombin, TNF-*α*, or endothelial growth factor (VEGF) treatments, stress fibers composed of actin and myosin play an important role in cell contraction and breaking down of the adherens junctions [48–50]. MLC is the light chain of myosin, and phosphorylation of MLC at either T18 or S19 is required for its interaction with actin [23]. Activation of RhoA/Rho kinase is considered to have a crucial role in the control of MLC phosphorylation and cytoskeletal rearrangements [22, 24, 46, 51–53]. LPA has been shown to activate RhoA/Rho kinase pathway in various cell types [25, 51, 54]. Our data indicate that an AM966-LPA1-RhoA/Rho kinase-MLC signaling pathway leads to cell contraction and adherens junctions disruption (Figure 6).

VE-cadherin is a fundamental component of adherens junctions in endothelium. By interacting with *α*-catenin, *β*-catenin, and p120, VE-cadherin links actin indirectly [20, 21]. Tyrosine phosphorylation of VE-cadherin increases vascular permeability [20]. There are nine tyrosine phosphorylation sites of VE-cadherin which may be phosphorylated in response to different stimuli [55]. VE-cadherin phosphorylation at Y685 is mediated by Src kinase, resulting in endothelial cell migration or hyperpermeability [56, 57], while phosphorylation at Y731 regulates the induction of leukocyte extravasation [58]. In our present study, phosphorylation of VE-cadherin at Y658 induced by AM966 leads to lung endothelial barrier dysfunction, consistent with several other studies where VEGF, polychlorinated biphenyl, and silver nanoparticles are the agonists [49, 59, 60]. LPA has been reported to increase permeability by regulating G*α*i/NF-kB signaling [61]. Here we show that this AM966-induced phosphorylation at Y658 is through the LPA1-G*α*12/13 pathway. FAK is a tyrosine kinase that plays different roles in barrier regulation. For example, FAK contributes to VEGF-induced barrier dysfunction [62, 63], while it has been also reported that downregulation of FAK protects barrier integrity [64]. In our study, though phospho-FAK is upregulated in response to AM966 stimulation, FAK kinase inhibitor did not reduce the level of VE-cadherin phosphorylation. In addition, FAK kinase inhibitor treatment enhances AM966-induced barrier disruption. The molecular mechanism by which AM966 induces phosphorylation of VE-cadherin is our focus in future studies.

AM966 has been known to bind to LPA1; however, the signaling pathway triggered by AM966 has not been revealed. This study is the first report to reveal that AM966 binds to LPA1 and triggers RhoA/Rho kinase-MLC and VE-cadherin phosphorylation pathways. This study indicates that LPA1 agonist and antagonist share LPA1/G*α*12/13 pathway regarding regulation of phosphorylation of MLC and VE-cadherin, while AM966 has no effect of activation of G*α*i-coupled LPA1 pathway.

## 5. Conclusion

In conclusion, our study, for the first time, shows an unexpected effect of AM966 on lung microvascular barrier disruption and underlying molecular mechanisms which are regulated through RhoA/Rho kinase/MLC and VE-cadherin (Figure 6). This is overlaps with LPA activity. Future study will be performed to examine the effect of AM966 and its related compounds on endothelial barrier integrity in preclinical murine models of human diseases.
